# Carnosine Mitigates Manganese Mitotoxicity in an In Vitro Model of Isolated Brain Mitochondria

**DOI:** 10.15171/apb.2019.034

**Published:** 2019-06-01

**Authors:** Vahid Ghanbarinejad, Asrin Ahmadi, Hossein Niknahad, Mohammad Mehdi Ommati, Reza Heidari

**Affiliations:** ^1^Pharmaceutical Sciences Research Center, Shiraz University of Medical Sciences, Shiraz, Iran.; ^2^Department of Pharmacology and Toxicology, School of Pharmacy, Shiraz University of Medical Sciences, Shiraz, Iran.

**Keywords:** Bioenergetics, Cirrhosis, Cognitive defects, Locomotor activity, Peptide, Manganese

## Abstract

***Purpose:*** Manganese (Mn) is a neurotoxic chemical which induces a wide range of complications in the brain tissue. Impaired locomotor activity and cognitive dysfunction are associated with high brain Mn content. At the cellular level, mitochondria are potential targets for Mn toxicity. Carnosine is a dipeptide abundantly found in human brain. Several pharmacological properties including mitochondrial protecting and antioxidative effects have been attributed to carnosine. The current study aimed to evaluate the effect of carnosine treatment on Mn-induced mitochondrial dysfunction in isolated brain mitochondria.

***Methods:*** Mice brain mitochondria were isolated based on the differential centrifugation method and exposed to increasing concentrations of Mn (10 µM-10 mM). Carnosine (1 mM) was added as the protective agent. Mitochondrial indices including mitochondrial depolarization, reactive oxygen species (ROS) formation, mitochondrial dehydrogenases activity, ATP content, and mitochondrial swelling and permeabilization were assessed.

***Results:*** Significant deterioration in mitochondrial indices were evident in Mn-exposed brain mitochondria. On the other hand, it was found that carnosine (1 mM) treatment efficiently prevented Mn-induced mitochondrial impairment.

***Conclusion:*** These data propose mitochondrial protection as a fundamental mechanism for the effects of carnosine against Mn toxicity. Hence, this peptide might be applicable against Mn neurotoxicity with different etiologies (e.g., in cirrhotic patients).

## Introduction

**Figure 1:**
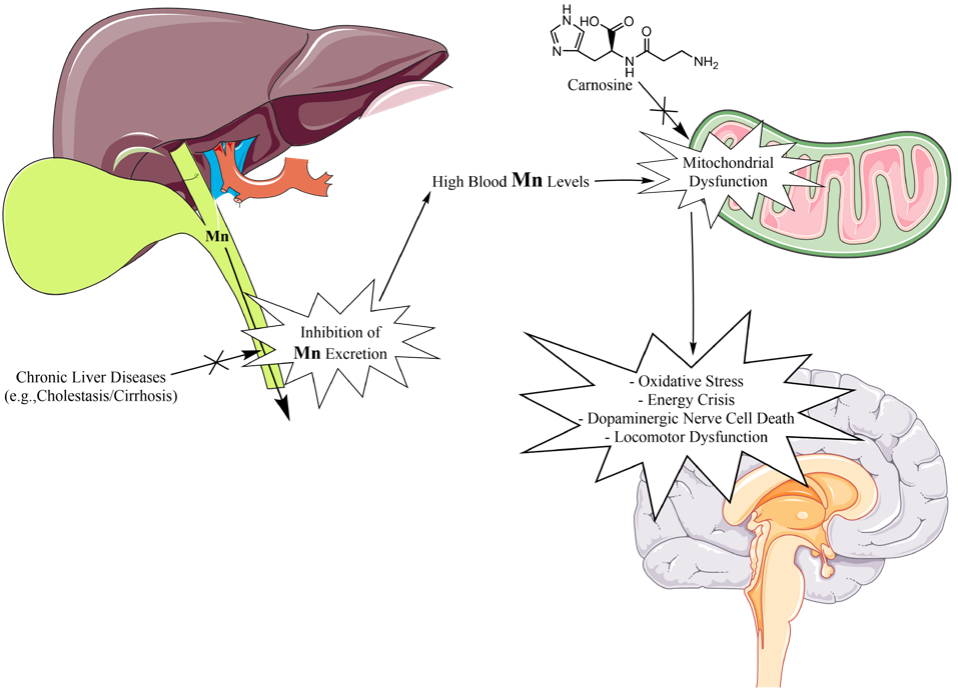
Schematic representation of the disturbances in manganese (Mn) excretion during cirrhosis, Mn-induced mitochondrial dysfunction, and the potential protective properties of carnosine.

Manganese (Mn) is an essential element incorporated in the structure of several vital enzymes.^1,2^ On the other hand, some pathological conditions could lead to Mn accumulation in the human body.^3^ The brain is the primary target of Mn toxicity.^4-7^ It has been found that increased body Mn levels led to severe neurological complications.^4,5^ Dopaminergic system is severely affected by Mn.^4-7^ Hence, Mn-induced neurotoxicity clinically appears as locomotor dysfunction resembles Parkinsonism (Figure 1).^4-7^

Mn is excreted in the bile (Figure 1).^8-11^ Therefore, any defect in Mn excretion could lead to serum and eventually brain high Mn levels (Figure 1).^8-11^ It has been found that liver failure and cirrhosis is associated with brain Mn accumulation.^8-11^ Cirrhosis-associated brain Mn accumulation could be involved in the pathogenesis of cirrhosis-related locomotor dysfunction (Figure 1).^8-11^

The cellular mitochondrion is a potential target of Mn toxicity.^12-17^ Mn is accumulated in the mitochondrial matrix through the calcium (Ca^2+^) transporters.^13,18,19^ It has been reported that Mn impaired cellular energy (ATP) metabolism and induced the release of cell death mediators form mitochondria (Figure 1).^5,17,18,20^

Carnosine is an endogenously found dipeptide which reaches very high concentrations in tissues such as skeletal muscle and the brain.^21^ Several pharmacological roles have been attributed to carnosine.^21-23^ This peptide is also widely evaluated for its neuroprotective properties.^22,24-27^ On the other hand, the mitochondrial protecting properties of carnosine have been mentioned in previous studies.^28-32^ Hence, it seems that carnosine provides its cytoprotection through regulation of cellular mitochondrial function.^28-31^

The current study was designed to evaluate the role of carnosine administration on Mn-induced mitochondrial injury in isolated brain mitochondria. Mice brain mitochondria were exposed to Mn (0.1 mM-10 mM) and carnosine (1 mM). Several mitochondrial indices including mitochondrial dehydrogenases activity, swelling, depolarization, and ATP content were assessed. The results might help to develop therapeutic options against Mn-induced CNS injury (*e.g.*, in cirrhotic patients).

## Material and Methods

### Chemicals

Carnosine was purchased from Sigma (St. Louis, MO, USA). 4,2-Hydroxyethyl,1-piperazineethanesulfonic acid (HEPES), 3-(N-morpholino) propane sulfonic acid (MOPS), Dimethyl sulfoxide (DMSO), D-mannitol, bovine serum albumin (BSA), thiobarbituric acid (TBA), 3-[4,5dimethylthiazol-2-yl]-2,5-diphenyltetrazolium bromide (MTT), Coomassie brilliant blue, Rhodamine 123 (Rh 123), Ethylene glycol-bis (2-aminoethyl ether)-N,N,N',N'-tetraacetic acid (EGTA), Sodium succinate, Hydroxymethyl aminomethane hydrochloride (Tris-HCl), and ethylenediaminetetraacetic acid (EDTA) were purchased from Merck (Darmstadt, Germany). All salts for preparing buffer solutions (analytical grade) were purchased from Merck (Darmstadt, Germany).

### Animals

Male BALB/c mice (20-30 g) were obtained from Animal Breeding Center of Shiraz University of Medical Sciences, Shiraz Iran. Animals were housed in plastic cages on wood-chip bedding at an ambient temperature of 23±2°C and relative humidity of ≈40%. Mice had free access to tap water and a standard rodent’s diet (Behparvar^®^, Tehran, Iran). Animals were handled according to the animal handling protocol approved by a local ethics committee at Shiraz University of Medical Sciences, Shiraz, Iran (01-36-15296).

### Brain mitochondria isolation

Mice brain mitochondria were isolated as previously described.^33^ Briefly, animals were anesthetized (ketamine/xylazine, 60/5 mg/kg, i.p) and their brain tissue was isolated and washed with ice-cold sodium chloride (Saline 0.9% w: v).^33,34^ The brain was homogenized in the mitochondria isolation buffer (70 mM mannitol, 220 mM Sucrose, 0.5 mM EGTA, 0.1% essentially fatty acid-free BSA, 2 mM HEPES, pH = 7.4) at a 10:1 w: v buffer to brain tissue ratio.^33^ Afterward, the tissue homogenate was centrifuged at 1000×*g* for 10 minutes at 4°C to remove intact cells and nuclei. The supernatants were further centrifuged (15 000×*g*, 4°C, 10 minutes) to precipitate the heavy membrane fractions (mitochondria).^35^ This step was repeated (at least three times) using fresh buffer medium to increase mitochondria yield. As mentioned, all manipulations for brain mitochondria isolation were done at 4°C or on ice to preserve mitochondrial intactness.^33^

### Mitochondrial swelling

Mitochondrial swelling was assessed based on the light scattering method as previously described.^33^ The isolated mitochondria (0.5 mg protein/mL) were suspended in the swelling buffer (125 mM Sucrose, 65 mM KCl, 10 mM HEPES, pH = 7.2). The light absorbance at λ = 540 nm was monitored (Constant temperature of 30°C) with a FLUOstar Omega^®^(BMG Labtech, Germany) multifunctional microplate reader.^33,36,37^ It is accepted that decreased light absorbance is coherent to an increase in mitochondrial volume.^37^ Therefore, as mitochondria are more swelled, the differences between light absorbance of two-time points are higher. The differences between the absorbance of samples were assessed at λ = 540 nm and reported as maximal mitochondrial swelling amplitude (ΔOD540 nm).^33^

### Mitochondrial depolarization

Mitochondrial uptake of the cationic fluorescence dye rhodamine 123 was used for the estimation of mitochondrial depolarization.^33,38-40^ For this purpose, the mitochondrial fractions (1 mg protein/mL) were incubated with rhodamine 123 (Final concentration 10 µM) in a buffer containing 125 mM Sucrose, 65 mM KCl, 10 mM HEPES, pH = 7.2 (20 minutes, 37°C, in the dark).^37,38^ Then, samples were centrifuged (15 000 g, 5 minutes, 4°C) and the fluorescence intensity of the supernatant was measured using a multifunctional fluorescent microplate reader (FLUOstar Omega^®^, BMG Labtech, Germany; λ _excitation_ = 485 nm and λ _emission_ = 525 nm).^33,41^

### Reactive oxygen species in isolated mitochondria

The fluorescent probe dichlorofluorescein diacetate (DCFH-DA) was used to evaluate the mitochondrial ROS measurement.^33,42,43^ Briefly, isolated brain mitochondria were incubated in the respiration buffer (125 mM Sucrose, 5 mM Sodium succinate, 65 mM KCl, 10 mM HEPES, 20 µM Ca^2+^, and pH = 7.2).^33^ Following this step, DCFH-DA was added (final concentration, 10 μM) and samples were incubated for 30 minutes (37°C, in the dark). Then, the fluorescence intensity of DCF was measured using a FLUOstar Omega^®^ (BMG Labtech, Germany) multifunctional fluorimeter (λ _excitation_ = 485 nm and λ _emission_ = 525 nm).^33^

**Figure 2:**
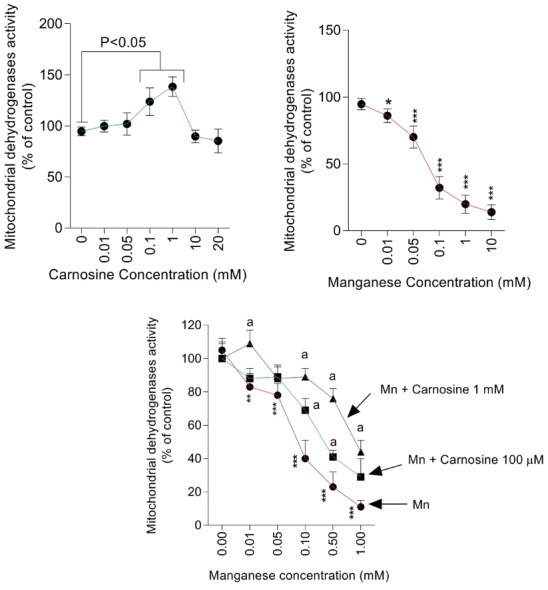
Mitochondrial dehydrogenases activity (MTT assay) in the presence of manganese (Mn) and carnosine. Data are given as mean ± SD (n = 8). Asterisksindicate significantly different as compared with control (0 mM manganese) group (**P < 0.01, ***P < .001).^a^ Indicate significantly different as compared with manganese (Mn) group (*P* < 0.001).

**Figure 3:**
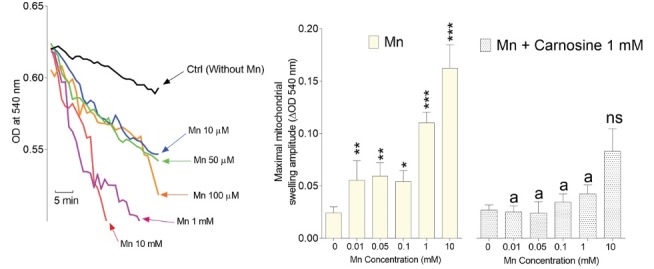
Manganese**(**Mn)-induced mitochondrial swelling in the presence of carnosine.

Data are given as Mean±SD (n = 8). Asterisks indicate significantly different as compared with control (Manganese 0 mM) (*P < 0.1, **P < 0.01, ***P< 0.001).^a^Indicates significantly different as compared with manganese alone (P < 0.05). ns: not significant as compared with similar dosage of the Mn-treated group.

**Figure 4:**
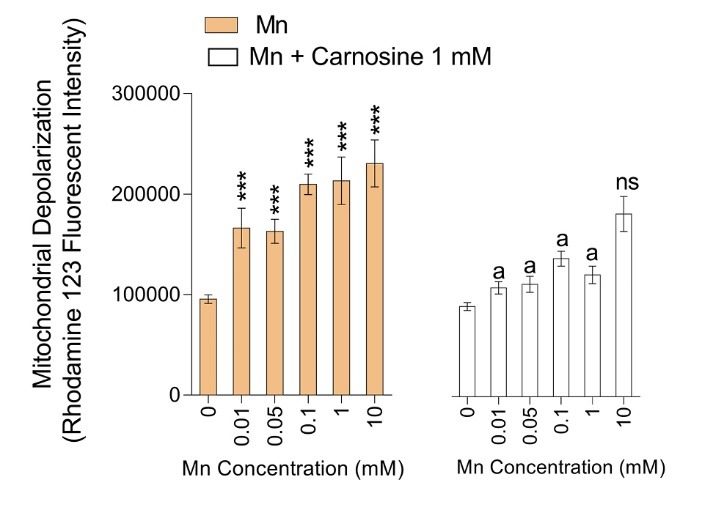
Mitochondrial depolarization in manganese (Mn)-treated brain mitochondria. Data are given as mean ± SD (n = 8).^***^ Indicates significantly different as compared with control (*P* < 0.001). ^a^ Indicates significantly different as compared with the Mn-treated group (*P* < 0.001). ns: not significant as compared with the similar dose of the Mn-treated group.

**Figure 5:**
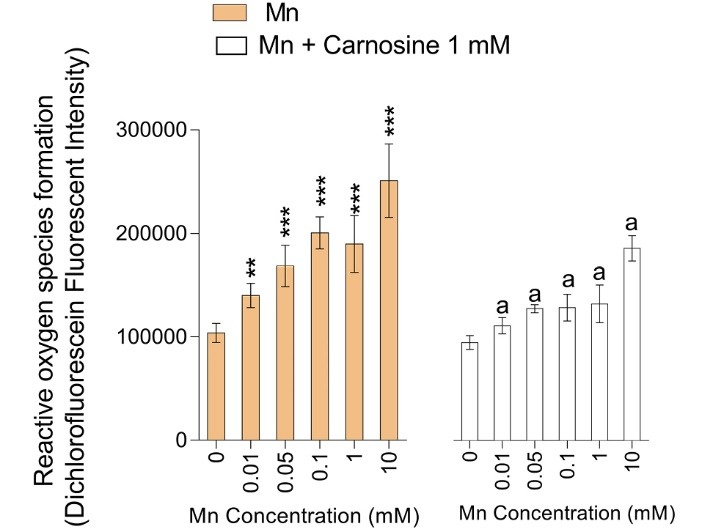
Mn-induced reactive oxygen species (ROS) formation in isolated mice brain mitochondria. Data are given as Mean±SD (n = 8). Asterisks indicate significantly different as compared with control (Manganese 0 mM) (**P < 0.01, ***P < 0.001). ^a^ Indicates significantly different as compared with the similar dose of Mn-treated group (*P* < 0.001).

**Figure 6:**
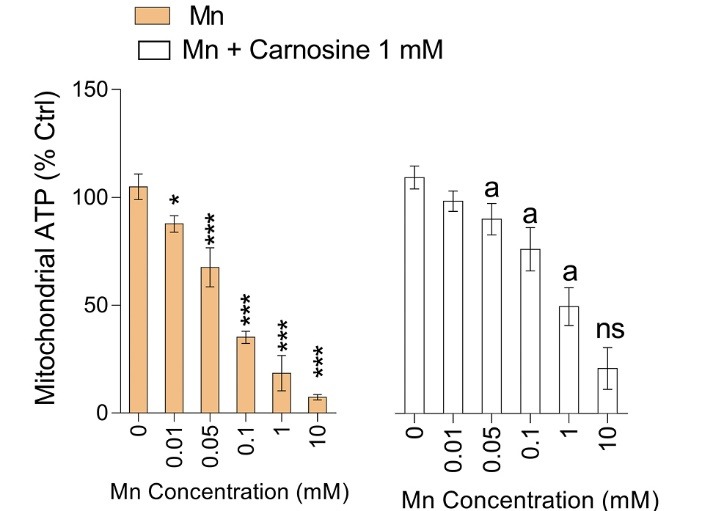
Mitochondrial ATP content. Data are given as Mean±SD (n = 8). Asterisks indicate significantly different as compared with control (Manganese 0 mM) (*P < 0.1, ***P < 0.001). ^a^ Indicates significantly different as compared with the similar dose of manganese alone (*P* < 0.05). ns: not significant as compared with the Mn-treated group.

### Mitochondrial ATP content

A luciferase-luciferin-based kit from Promega (ENLITEN^®^) was used to assess mitochondrial ATP content.^44^ Samples and buffer solutions were prepared based on the kit instructions, and the luminescence intensity of samples was measured at λ = 560 nm using a FLUOstar Omega^®^ (BMG Labtech, Germany) multifunctional microplate reader. For standardization of data, samples protein concentrations were determined by the Bradford method.^45^

### Statistical analysis

Data are given as the mean±SD. Data comparison was performed by the one-way analysis of variance (ANOVA) with Tukey’s multiple comparison test as the post hoc. Differences were considered statistically significant when *P* < 0.05.

## Results

Brain mitochondria exposure to Mn was associated with decreased mitochondrial indices of functionality. Severe decrease in mitochondrial dehydrogenases activity was detected in Mn-treated mitochondria (Figure 2). It was found that pre-incubation of mice brain isolated mitochondria with carnosine (1 mM) significantly prevented Mn-induced decrease in mitochondrial dehydrogenases activity (Figure 2). As 100 µM concentration of carnosine was not effective against a high dose of Mn (Figure 2), higher concentration (1 mM) of the peptide was selected for further assessments.

Significant mitochondrial permeability and swelling were evident in Mn-exposed isolated brain mitochondria as assessed by the light scattering method (Figure 3). On the other hand, it was found that carnosine treatment (1 mM) significantly mitigated Mn-induced mitochondrial permeabilization and swelling (Figure 3).

The collapse of mitochondrial membrane potential was another adverse effect of Mn on isolated mice brain mitochondria (Figure 4). Mn-induced mitochondrial depolarization was revealed by a decrease in mitochondrial capacity of rhodamine 123 capture (Figure 4). It was found that carnosine treatment (1 mM) significantly prevented Mn-induced mitochondrial depolarization (Figure 4).

Evaluation of reactive oxygen species (ROS) in Mn-treated mice brain mitochondria revealed a significant increase in DCF fluorescent intensity (Figure 5). On the other hand, carnosine administration (1 mM) significantly ameliorated Mn-induced ROS formation in isolated brain mitochondria (Figure 5).

Significant depletion of mitochondrial ATP content was also detected in Mn-treated mice brain mitochondria (Figure 6). It was found that carnosine (1 mM) supplementation preserved mitochondrial ATP content at a higher level in comparison with Mn-exposed group (Figure 6).

## Discussion

The primary object of the current investigation was to search the effect of carnosine treatment on Mn-induced mitochondrial dysfunction. The data obtained from this study might help to develop therapeutic options against cirrhosis and its associated complications as well as Mn-induced neurotoxicity with different etiologies.

Several liver diseases including chronic liver injury and cirrhosis are associated with brain tissue Mn deposition (Figure 1).^9,46,47^ Environmental Mn exposure could also result in neurodegenerative disorders.^48,49^ Mn is a neurotoxic trace element which adversely affects locomotor and cognitive function.^47,50^ Severe changes in the concentration of different neurotransmitters have been documented in Mn-exposed animals.^51^ On the other hand, at the cellular level mitochondria are potential targets of Mn toxicity.^4,5,15,16,20,52-54^ Hence, mitochondria protecting agents might serve as potential therapeutic options against Mn cytotoxicity (Figure 1). In the current study, Mn exposure concentration-dependently enhanced mitochondrial dysfunction. On the other hand, it was found that carnosine (1 mM) supplementation efficiently mitigated Mn-induced impairment of mitochondrial function in isolated mice brain mitochondria.

It is well-established that Mn accumulates in the mitochondrial matrix, interrupts oxidative phosphorylation, and inhibits energy (ATP) metabolism.^13,18,19^ On the other hand, the alteration in mitochondrial permeability transition induced by Mn leads to mitochondrial swelling and release of several cell death mediators from this organelle.^13,18,19^ Inhibition of mitochondrial electron transport chain has also been mentioned in Mn-exposed mitochondria.^13,18,19^ Therefore, protecting cellular mitochondria could serve as a potential therapeutic strategy against Mn cytotoxicity (Figure 1).

The involvement of carnosine in the regulation of mitochondrial function has been previously mentioned in different experimental models.^28,29,31,55^ Carnosine regulates mitochondrial matrix pH, preserves mitochondrial membrane potential, increases the activity of the respiratory chain complexes, and enhances mitochondrial energy production.^29,56-59^ The anti-apoptotic properties of carnosine also have been mentioned in several investigations.^30,58,59^ All these properties make carnosine as an effective mitochondrial protecting agent and indicate that this peptide could be a potential safe therapeutic option against a wide range of mitochondrial-linked complications (Figure 1).

Our previous findings mentioned that carnosine administration efficaciously alleviated chronic liver injury and its associated complications.^30,60^ The motor deficit is one of the significant features of cirrhosis and chronic hepatic encephalopathy.^61,62^ Muscle stiffness, poor muscle coordination, rigidity, and tremor are observed in cirrhotic patients.^61^ On the other hand, a several-fold increase in the plasma and brain Mn level of cirrhotic patients has been established.^11,63^ Hence, increased brain Mn levels could be associated with CNS damage in cirrhotic patients (Figure 1). As mentioned, brain Mn accumulation is a complication related to liver failure and cirrhosis.^11,63^ The results of this study suggest that carnosine not only provide beneficial effects against cirrhosis, hyperammonemia, and tissue fibrosis,^30,60^ but also might prevent Mn-induced mitochondrial dysfunction and protect the CNS in cirrhosis.

Mitochondria are the most critical intracellular sites of ROS formation.^64^ It has been well-established that superoxide anion (O_2_^.^-) and hydrogen peroxide (H_2_O_2_) are produced during mitochondrial respiration.^64^ On the other hand, mitochondria-mediated ROS formation could be enhanced by xenobiotics.^65,66^ The data obtained in the current study revealed that exposure of brain mitochondria to Mn hasten ROS formation in this organelle (Figure 4). Meanwhile, carnosine (1 mM) mitigated Mn-induced ROS formation (Figure 4). The antioxidant and ROS scavenging properties of carnosine have repeatedly been mentioned in previous investigations.^22,23,26,55^ It has been established that this peptide possesses antioxidant effects in different experimental models.^22,23,25,26,55,60^ Carnosine also efficiently scavenges reactive end products of oxidative stress.^67^ Hence, another important mechanism of protective properties of carnosine could be mediated through its antioxidant properties and decrease of mitochondria-born ROS (Figure 1).

The data obtained in the current study might help developing safe, protective agents against Mn neurotoxicity which is involved in the pathogenesis of cirrhosis-associated CNS complications. Indeed, further research on the effect of carnosine on mitochondrial respiratory complexes as well as the mPT components will enhance our understanding of the mitochondrial protecting properties of this naturally occurring peptide. On the other hand, carnosine could be considered as a promising pharmacological intervention in attenuating Mn-induced neurotoxicity with different etiologies (e.g., cirrhosis).

## Ethical Issues

Animals were handled and used according to the animal handling protocol approved by a local ethics committee at Shiraz University of Medical Sciences, Shiraz, Iran (#01-36-15296).

## Conflict of Interest

The authors declare that they have no conflicts of interest.

## Acknowledgments

This work was supported by the Pharmaceutical Sciences Research Center and the Office of Vice Chancellor of Research Affairs of Shiraz University of Medical Sciences (grant No. 15296/15295/14210/12832).
